# Retraction: Matrine alleviates lipopolysaccharide-induced intestinal inflammation and oxidative stress via CCR7 signal

**DOI:** 10.18632/oncotarget.28119

**Published:** 2023-12-01

**Authors:** Guojun Wu, Wenhong Zhou, Junfeng Zhao, Xiaohua Pan, Yongjie Sun, Hao Xu, Peng Shi, Chong Geng, Ling Gao, Xingsong Tian

**Affiliations:** ^1^Department of Breast and Thyroid Surgery, Shandong Provincial Hospital Affiliated to Shandong University, Jinan 250021, Shandong, P. R. China; ^2^Department of Nursing, Shandong Provincial Hospital Affiliated to Shandong University, Jinan 250021, Shandong, P. R. China; ^3^Traffic Police Department, Jinan Public Security Bureau, Jinan 250021, Shandong, P. R. China; ^4^Scientific Center, Shandong Provincial Hospital Affiliated to Shandong University, Jinan 250021, Shandong, P. R. China; ^*^These authors contributed equally to this work

**This article has been retracted:** The authors have been unable to replicate their published results and are therefore no longer confident in the conclusions of their research. In light of this, and with the agreement of all authors, Oncotarget has decided to retract this paper.

Original article: Oncotarget. 2017; 8:11621–11628. 11621-11628. https://doi.org/10.18632/oncotarget.14598

